# Efficacy and safety of using a warming needle for persistent allergic rhinitis: study protocol for a randomized controlled trial

**DOI:** 10.1186/s13063-016-1432-z

**Published:** 2016-06-30

**Authors:** Yuxiu Sun, Hong Zhao, Yongming Ye, Wenbin Nie, Wenjing Bai, Jia Liu, Sinuo Li, Fang Wang, Mingjuan Han, Liyun He

**Affiliations:** Guang’anmen Hospital, China Academy of Chinese Medical Sciences, Beijing, 100053 China; Institute of Acupuncture and Moxibustion, China Academy of Chinese Medical Sciences, Beijing, 100700 China; Mentougou Hospital of TCM, Beijing, 102300 China; China Academy of Chinese Medical Sciences, Beijing, 100700 China; Hubei University of Chinese Medicine, Wuhan, 430061 China

**Keywords:** Acupuncture, Warm needling, Persistent allergic rhinitis, RCT, Study protocol

## Abstract

**Background:**

Many previous studies have shown the potential therapeutic effect of acupuncture for allergic rhinitis. Most of these studies, however, were limited by the short duration of observations and lack of sham acupuncture as the control group. Our preliminary experiments showed that the use of a warm needling achieved a much more persistent effect in the treatment of allergic rhinitis (AR) compared with simple acupuncture therapy. Hence, we have designed a multicenter, randomized controlled trial (RCT) in which the first-line medication loratadine will be used as the control group, and the effect of warm needling therapy will be evaluated through long-term observation.

**Methods/design:**

The trial is designed as a multicenter, parallel-group, randomized, single-blinded (outcome assessors), non-inferiority trial. A total of 98 patients with persistent AR will be randomly assigned into two groups. Patients in the treatment group will be treated with warm needling at GV14 and acupuncture at EX-HN3, ST2, LI20, EX-HN8, GV23, LU7, LU5 and LI4 three times a week, for a total of 4 weeks. Patients in the control group will be treated with oral loratadine 10 mg/day for 4 weeks. The primary outcome will be the change in the Total Nasal Symptom Score (TNSS) from baseline to that at 6 months after treatment during the follow-up period. The secondary outcomes will include the Total Non-nasal Symptom Score and the Rhinoconjunctivitis Quality of Life Questionnaire, changes in the TNSS from baseline to that at 2 and 4 weeks during treatment, and 3 months after treatment during the follow-up period. Outcomes will be measured at 2 and 4 weeks, and 3 and 6 months after treatment. Any side effects of treatment will be observed and recorded.

**Discussion:**

We expect that the study results will provide evidence to determine the effects of warm needling compared with loratadine. Our final goal of the study is to evaluate the difference in the short-term and long-term effects between the two therapeutic methods, especially the long-term effect of warm needling.

**Trial registration:**

ClinicalTrials.gov NCT02339714. Registered on 14 January 2015.

**Electronic supplementary material:**

The online version of this article (doi:10.1186/s13063-016-1432-z) contains supplementary material, which is available to authorized users.

## Background

Allergic rhinitis (AR) is a symptomatic nasal disorder resulting from an IgE-mediated immunological reaction to allergen exposure [1]. AR is a worldwide health problem affecting people of all ages. The reported incidence of AR is 11.8–46 % worldwide [2, 3] and 11.1–19.1 % in China [4, 5]. AR is classified into intermittent allergic rhinitis (IAR) and persistent allergic rhinitis (PAR) according to the time of exposure. The major symptoms of AR include rhinorrhea, nasal itching, nasal obstruction, and sneezing. These symptoms can resolve spontaneously or with treatment. AR may cause disruption in sleep cycles and emotional balance, and impair daily activities [6]. Untreated AR can also trigger other diseases such as bronchial asthma, sinusitis, nasal polyps, otitis media, and allergic conjunctivitis, resulting in significant psychological disturbance and economic burden [7].

The current mainstream medical management of AR primarily includes allergen avoidance, pharmacotherapy, immunotherapy and patient education. These treatments, however, do not always provide complete relief of symptoms and are not usually cost-effective, and like other medications they come with undesirable side effects [8, 9]. Although newer-generation medications have been developed, the issue of side effects has not been resolved [10]. Furthermore, pharmacotherapy only provides quick symptom relief in the case of PAR and becomes less effective in recurrent cases due to the development of medication tolerance [11]. Immunotherapy can reduce the natural duration of AR and provide symptom control; however, it can cause a local or systemic anaphylactic reaction [12]. Therefore, a safe and long-term effective treatment modality for AR is needed to provide symptom relief, regulate immune system function, decrease frequency and severity of AR recurrence, and prevent the condition from progressing.

The results from our previous international, multicenter clinical study in collaboration with South Korea revealed that 4-week long, active acupuncture treatment was significantly better at reducing AR symptoms compared with sham acupuncture and non-active treatment [13]. Furthermore, AR symptoms continued to reduce in the subsequent 4-weeks of follow-up, indicating the residual effect of acupuncture. Previous studies have demonstrated that moxibustion has a regulative effect on immune system function [14, 15]. Consequently, we have optimized the intervention scheme to warm needling. There are two types of warm needling, the one we used consists of placing an ignited moxa stick on the handle of the needle after insertion. Our hypothesis is that warm needling will achieve equal or better long-term symptom relief of AR and reduce its recurrence.

In previous studies of immunotherapy and pharmacotherapy effects on AR, the placebo control group treatment provided over 50 % symptom relief [16]. In previous clinical studies of AR treated with acupuncture therapy, placebo acupuncture was widely adopted as the control group. In this study, however, we have selected western medicine as the positive control instead of placebo acupuncture. Oral antihistamines are usually the first-line medication for AR symptom relief. Loratadine, as a second-generation antihistamine agent, has a strong selective antagonistic effect on the peripheral histamine H1 receptors, providing long-lasting effect without sedating the central nervous system. Not only is loratadine used in relieving and preventing AR-associated symptoms such as rhinorrhea, itching, sneezing, nasal blockage, and ocular symptoms [17, 18], it is also widely used in the treatment of other allergic diseases. After a detailed evaluation of the desired and the adverse effects, we chose loratadine as the intervention for the control group.

We have designed a multicenter, randomized controlled trial (RCT) for patients with PAR, using oral loratadine as control. The purpose of this trial is to answer two questions: (1) is the effect obtained by warm needling equal to or better than that achieved by loratadine? and (2) is the treatment of warm needling safe for patients with PAR?

The study was registered on ClinicalTrials.gov (NCT02339714).

## Methods

### Study design

The trial is designed as multicenter, parallel-group, randomized, single-blinded (outcome assessors), non-inferiority trial, comparing the differences between warm needling therapy and loratadine for PAR.

We will recruit 98 patients with PAR from three hospitals in China. According to their group assignment, patients will receive either 12 sessions of warm needling or the oral medication loratadine for 4 weeks.

**Fig. 1:**
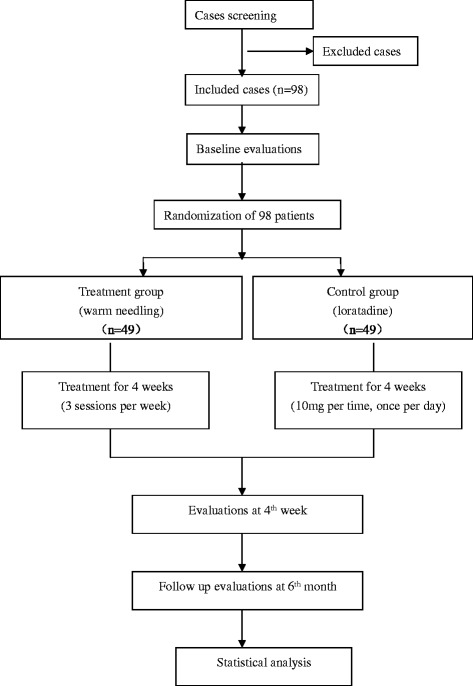
Flowchart of the study design

The study design flowchart is presented in Fig. 1, and the study design schedule is shown in Table 1.

**Table 1:** Study design schedule

Period	Screening	Baseline	Treatment (W1–4)		Follow-up (W5 − M6)	
Time	W − 1	W0	W2	W4	M3	M6
Eligibility	X					
Demography and medical history	X					
Physical examination	X					
Informed consent	X					
TNSS	X	X	X	X	X	X
TNNSS	X	X	X	X	X	X
RQLQ	X	X	X	X	X	X
Adverse event			X	X		
Compliance			X	X	X	X

*RQLQ* Rhinoconjunctivitis Quality of Life Questionnaire, *TNSS* Total Nasal Symptom Score, *TNNSS* Total Non-nasal Symptom Score

### Study population

#### Trial location

We will recruit patients from three hospitals:

1. Guang’an Men Hospital of China Academy of Chinese Medical Sciences (CACMS)
2. Acupuncture and Moxibustion Hospital of CACMS
3. Mentougou Hospital of Traditional Chinese Medicine (TCM).

The three research centers are all situated in the capital of China.

#### Inclusion criteria

Participants will be eligible if they meet all of the following criteria: (1) are aged 18–60 years, (2) meet the classification criteria of moderate to severe PAR according to the Allergic Rhinitis and its Impact on Asthma (ARIA) guidelines, (3) exhibit at least one positive result on the allergy skin prick reaction test, (4) a 7-day mean Total Nasal Symptom Score (TNSS) ≥4 before treatment, and (5) agree to participate in the study willingly and voluntarily and sign the informed consent.

#### Exclusion criteria

Patients will be excluded if they have any of the following conditions: (1) suffer from other diseases that have an influence on PAR symptoms, (2) a moxa smoke allergy, or a severe needle phobia, (3) have experienced an acute paranasal sinusitis or respiratory tract infection within the previous 2 weeks, (4) a history of chronic paranasal sinusitis or chronic nasal sinusitis as shown by paranasal sinus computed tomography examination, or history of organic nasal cavity disease or nasal cavity surgery, (5) radiographic signs of pulmonary inflammation, (6) paroxysmal respiratory diseases such as asthma, (7) have taken antihistamines (including H1 and H2 receptor blockers), glucocorticoids, decongestants, mast cell membrane stabilizers, leukotriene antagonists, antibiotics or other prohibited drugs (including nasal, oral, or ophthalmic usage) or herbal preparations for AR within the last 2 weeks, (8) have undergone specific immunotherapy or systematic hormone therapy within the year prior to enrollment, (9) have received acupuncture and moxibustion, nasal inhalation of herbal medicine, physical therapy or other external treatment for AR within the last 2 weeks, (10) suffer from a mental disorder, malignant tumor, severe cardiovascular disease, severe endocrine disorder, tuberculosis, hepatitis, or serum creatinine or aspartate transaminase/alanine transaminase (AST/ALT) above twice the normal level, and (11) be unable to cooperate in the trial.

### Study procedures

#### Recruitment

In order to achieve the target enrollments of participants, clinical recruitment flyers were placed in three hospitals. Clinical recruitment staff will be in charge of the enrollment process of the 98 participants with PAR. Participants will be screened based on the inclusive and exclusive criteria, be briefed on the purpose, procedures, treatments and possible risks of the trial, and be clearly inform of their rights to discontinue participation in the clinical study. The screening process takes about 1 week, requiring the participants to fill in questionnaires on symptoms during the 1-week period. Upon participants signing off the consent forms, the staff will then assign the recruited patients to different intervention groups.

#### Randomization

The patients will be randomly assigned in a 1:1 ratio to the warm needling group or the loratadine group. The randomization sequence was computer-generated by the Clinical Evaluation Center of CACMS and the corresponding group name will be kept in a sealed and consecutively numbered security envelope. The envelope will be kept by the recruitment staff of the three centers. After all patients have signed the informed consent form, the envelopes will be opened. The patients will receive either warm needling treatment or oral medication of loratadine treatment according to their assigned group.

#### Blinding

Since the acupuncture group and the medication group have different characteristics regarding intervening measures and approaches, it is not feasible to blind both the operators and patients. Instead we adopted the single-blinded trial where both the outcome evaluator and statistical analyst will be blinded to the groupings and they will not be involved in any part of the treatments during trial to ensure that there no form of bias results. At the end of the observation period, un-blinding is permissible only after database blinding approval and data block.

#### Follow-up

Upon completion of the 4-week treatment period, there is a further 6-month follow-up period. The research staff will continue to follow the participants via telephone communication and short messaging service (SMS). The frequency of follow-up is monthly and we will keep a record of the assessment of symptoms and medication compliance or changes. In the event of participants discontinuing or deviating from intervention protocols, the study staff will record the reasons for this and their medications and then exclude the participants from the study, and the latest outcome data including symptoms and frequency of the attacks.

### Intervention

#### Treatment group

Licensed acupuncture physicians will provide warm needling treatment three times a week for 4 weeks. The acupuncture needles are disposable and sterile and measure 0.25 × 25 mm and 0.25 × 40 mm (Suzhou Medicine Equipment Co., Ltd., Suzhou, China.). The moxa stick (Nanyang Lvying Moxibustion Product Co., Ltd., Nanyang, China) will be pure moxa measuring 12 mm in diameter and 15 mm in length. The acupuncture points used will be *Dazhui* (GV14), *Yintang* (EX-HN3), *Sibai* (ST2), *Yingxiang* (LI20), *Shangyingxiang* (EX-HN8), *Shangxing* (GV23), *Lieque* (LU7), *Chize* (LU5), and *Hegu* (LI4), which will be located according to the World Health Organization (WHO) International Standard Acupuncture Points. Patients will be in the seated position during treatment sessions. After routine skin sterilization, needles will be inserted using a neutral reinforcing and reduced manipulation technique. Each needle will be rotated until the arrival of *qi*. After the needle is obliquely inserted at *Dazhui* (GV14), the moxa stick will be ignited and attached to the needle handle with the ignited end toward the skin 1–1.5 cm from the skin surface. When the first moxa stick has burnt out, the ashes will be removed and the second stick will be replaced following the same procedure. In total, two moxa sticks are used. All needles will be retained for 30 min. Patients will feel a sense of local warmth, and the surrounding skin will become mildly red during the procedure. All of the practitioners have had more than 1 year of clinical acupuncture experience since completing a 5-year-long college of TCM training. The procedure for conducting warm needling therapy has been standardized in each center by training in advance (see Table 2).

**Table 2:** Acupoints and needling procedure

Acupoints	Angle and direction	Depth (mm)
*Dazhui* (GV14)	Oblique insert downwards, then penetrate towards the acupuncture point *Shenzhu* (GV12)	26–32
*Yintang* (EX-HN3)	Transversely, downward	13
*Sibai* (ST2)	Transversely, towards the nose	8
*Yingxiang* (LI20)	Transversely, towards the root of nose	5
*Shangyingxiang* (EX-HN8)	Transversely, towards the nose	8
*Shangxing* (GV23)	Transversely, downward	8
*Lieque* (LU7)	Transversely, upward	8
*Chize* (LU5)	Perpendicular, upward	13–21
*Hegu* (LI4)	Perpendicular to the skin	21–26

Precautions: in the process of inserting the ignited moxa stick into the needle handle, acupuncture physicians hold the junction of the needle handle and needle body with one hand while inserting the moxa stick into the needle at *Dazhui* (GV14) gently with the other hand so as to prevent skin burns. After attaching the moxa stick to the needle handle, acupuncture physicians ensure the stick is fully fixed on the needle handle to prevent it from falling on to the skin surface. The patient will be closely monitored during the entire procedure by research assistants to ensure safety and that any accident is handled in a timely manner.

#### Control group

Loratadine (Shanghai Xianlingbaoya Medicine Co., Ltd., Shanghai, China) will be prescribed for oral administration at a dosage of 10 mg/day in the morning. Each treatment cycle will be for four consecutive weeks.

#### Concomitant care and intervention

During the treatment and follow-up period, the subjects are advised to avoid antigens. The participants are prohibited from taking medications such as oral and intranasal antihistamines, intranasal steroids, intranasal decongestant, antibiotics or oral leukotriene receptor antagonists. Only for patients with severe symptoms are intranasal steroids allowed to be used. The type of medicine, dosage and usage will be recorded in the diary card for analysis. For the other complicated chronic diseases, the patients must continue taking their routine medication and other therapies. The research staff will record the names of those diseases and the names of medications and therapies in the case report.

#### Discontinuation of the intervention

The intervention is to be terminated in the cases of severe adverse events, withdrawal of participants by their own wish and unpermitted medication use.

### Outcome measures

It has been found that similar outcomes have been applied in the previous studies on PAR, such as the Total Nasal Symptom Score (TNSS), the Total Non-nasal Symptom Score (TNNSS) [19, 20], the Rhinoconjunctivitis Quality of Life Questionnaire (RQLQ) [21–23], the Nasal Provocation Test [24], IgE level, eosinophil count [25], and Visual Analog Scale (VAS) [26]. The TNSS, TNNSS, and RQLQ have been adopted especially in this study due to their wide application.

#### Primary outcome measure

The average change in the TNSS will be measured blindly by comparing the baseline score in each group with that at 6 months after treatment during the follow-up period. The TNSS is determined in terms of the severity of rhinorrhea, nasal itching, nasal obstruction, and sneezing.

#### Secondary outcome measures

The average change in the TNSS will be measured by comparing the baseline score in each group with that at 2 and 4 weeks during treatment, 3 months after treatment during the follow-up period. The average change in the TNNSS will be reviewed blindly by comparing the baseline score in each group with that at 2 and 4 weeks, and 3 and 6 months after treatment. The TNNSS is determined in terms of the supplementary symptoms such as post-nasal discharge, tearing, nasal or ocular itching, nasal or maxillary pain, and headache. The RQLQ will be used to observe the effect on patients’ quality of life; this will also be evaluated at 2 and 4 weeks, and 3 and 6 months after treatment.

All outcome readings will be scored on quantitative scales, and will be summarized as mean values and standard deviation.

#### Safety evaluation of warm needling therapy

Adverse events such as burns, skin rash, pain or other adverse reactions will be recorded in detail, including time of occurrence, duration of symptoms, severity, management measures, time of adverse reaction disappearance, and any apparent correlation to the administration of warm needling therapy and/or medication. Clinical physicians will decide whether to cease the treatment according to the severity of the adverse events.

**Fig. 2:**
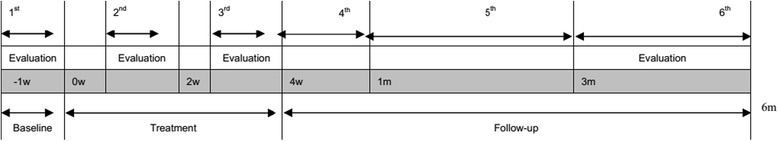
The total study period and the evaluation time points

The total study period and the evaluation time points, see Fig. 2.

### Data collection and management

The research staff is responsible for the collection of baseline characteristic data and the allergen test results during the screening period. The baseline variables will include: center location, age, gender, highest education level achieved, employment, diagnosis, nasal mucosal examination result, allergen examination result, typical symptoms, occurrence time of symptoms, time of recurrence or aggravation, accessory examination of nasal mucosa and nasal sinus, family history, relevant diseases (allergic asthma, allergic conjunctivitis), other relevant clinical disorders (such as hypertension, diabetes, and cardiac arrhythmia), as well as the questionnaires such as the TNSS, the TNNSS, and the RQLQ.

Participants are required to record in the daily diary the symptoms of AR and intake of any other medications during this study period. Outcome evaluators will examine the outcomes at baseline, 2 weeks (within treatment), 4 weeks (end of treatment), 3 months (within follow-up), and 6 months (end of follow-up). Data on nasal symptoms, non-nasal symptoms, and patients’ quality of life will be collected.

Data monitoring and management will be performed by the Clinical Evaluation Center of CACMS every 3 months. Two assistants will enter all data into an electronic database by double data entry. The statistical manager will be responsible for source data organizing, coding, range checking for data values and converting data to ensure data quality.

### Statistical analysis

#### Sample size calculation

The sample size calculation was performed with SAS 9.3 software (SAS Institute Inc., Cary, NC, USA) in the Clinical Evaluation Center of CACMS. The mean change in TNSS before and after treatment was used as the indicator for the efficacy evaluation in the calculation of the sample size. Results from our previous studies showed that the mean TNSS change was 4.90 ± 3.54 after acupuncture treatment [27] and 3.4 ± 1.0 after loratadine treatment [28]. Thus, we performed a non-inferiority test to calculate the appropriate trial sample size with 80 % power, alpha of 0.05, and an acceptable delta of 0.2. The results showed that a clinically important difference can be detected by a sample size of at least 39 in each group. This number was then increased to 49 per group (total of 98) to allow for a predicted 20-% dropout rate.

#### Statistical methods

The statistical analysis will be performed with SPSS 17.0 software (SPSS Inc., Chicago, IL, USA) in the Clinical Evaluation Center of CACMS. We will perform baseline adjusted analyses for the following variables (the center and severity), and the baseline value of the corresponding outcomes will be assessed. The data analyses will be conducted on an intention-to-treat population. Descriptive statistics will be used to compare the baseline measures and patients’ characteristics between the two groups. Regarding the primary and secondary outcome measures, the two-sample *t* test or Wilcoxon rank sum test will be used to compare the differences between the two groups from baseline to the end of treatment (*p* < .05 will be considered statistically significant). If an imbalance occurs in baseline characteristics between the two groups, ANCOVA will be applied.

#### Missing data and sensitivity analysis

The data used in the main statistical analysis should be collected in the fourth week of the treatment and at the half-year follow-up. In order to avoid missing data, we will provide an economic compensation for participants who have finished the trial and provided the completed data. The investigators have a wealth of experience in managing patients and collecting data in previous trials. They record the contact information of participants and keep in touch with them through a variety of means during the treatment and follow-up periods.

If the data are not obtained, we will record the time and reason for the missing data and will analyze the assumed missing data mechanism. For these missing data, we will use the multiple imputation adjustment approach. After the main analysis, we will perform a sensitivity analysis for the various datasets to enable us to judge the impact of missing data on our results.

A fully specified statistical analysis plan will be written independently.

### Monitoring

All staff involved in the study must receive training in advance. The training includes the use of randomized envelopes, the method for filling out case report forms, details of the warm needling procedure, the use of scales, the method for the patients to fill in the diary, and follow-up visit skills. Staff have been examined after training to ensure the consistency of warm needling treatment and the evaluation method.

A special research team from the Clinical Evaluation Center of CACMS, independent from investigators and the sponsor, will perform external monitoring of this study in the three hospitals every 3 months during the study to ensure the authenticity of the data. An advisory board will follow the trial and give advice when necessary. The data monitoring committee (DMC) has been established independent of the sponsor and there is no conflict of interest. It is responsible for monitoring the trial progression and guaranteeing the patients’ safety. We have not specified interim analyses and stopping plans of the trial, but if the DMC requests interim analyses, these will be supplied.

In order to improve adherence of the intervention, we will deliver the medicine once a week to the control group, and provide free treatment in the treatment group for 4 weeks. At the end of treatment and follow-up, we will supply a monetary compensation to the participants to ensure that treatment and follow-up run to schedule.

### Ethics and dissemination

#### Ethics

This study was designed in accordance with Chinese corresponding regulations and the principles of the 1996 Declaration of Helsinki, and has been approved by the Ethics Committee of the Institute of Acupuncture and Moxibustion of CACMS (201410301), Mentougou Hospital of TCM (201501151), and the Ethics Committee of Guang’anmen Hospital, CACMS (2015EC043).

The research assistants will fully explain the purpose, procedure, treatment and possible risks of the trial, and clearly inform the patients of their right to discontinue the study. The participants are required to sign the informed consent forms before the trial. The research assistants are in charge of the storage of all the informed consent forms.

This is the protocol of the second version (v2.0 dated 26 April 2015). On the basis of the first version, the exclusive criteria have been modified slightly in the second version. All protocol versions have been submitted to the ethics committees of the three hospitals. Research ethics committee review board approvals are kept with the ethics committees of the three hospitals.

Additionally, the participant data and biological specimens obtained in this ongoing research will not to be used for other purposes. All of the involved personal information, such as the informed consent form, the case report form, the patient diary, and the physical examination findings are stored by category in a specific file cabinet before, during, and after the trial to protect confidentiality. The sponsor, statistical manager, and monitoring manager will have access to the final trial dataset and disclosure of contractual agreements.

After the trial, if the participants are willing to continue treatment, we will provide corresponding therapies. For those who suffer harm from trial participation, we will provide a certain amount of economic compensation or free treatment (especially for those with adverse events).

#### Publications and dissemination

After completing data analysis, publications will be planned in Chinese and English versions. Regardless of the findings, we plan to disseminate all of the trial results in conferences or publications.

All of the staff who participated in the organization, implementation, data management and data statistical analysis will be affirmed in the authorship and we do not intend to use any professional writers.

There is no plan to allow public access to the full protocol, participant dataset, or statistical code. But, if necessary, individuals can gain access to the full protocol through the Ethics Committee of the Institute of Acupuncture and Moxibustion of CACMS.

This protocol was written following the Standard Protocol Items: Recommendations for Interventional trials (SPIRIT) checklist (see Additional file 1). The future report will follow the Consolidated Standards of Reporting Trials (CONSORT) guidelines [29], Revised Standards for Reporting Interventions in Clinical Trials of Acupuncture [30], and the extension of CONSORT for reporting non-inferiority randomized trials [31].

## Discussion

The results of this study are expected to provide reliable evidence for the long-term efficacy of warm needling in the treatment of PAR.

This trial is designed as a non-inferiority trial. In our previous study comparing acupuncture and sham-acupuncture (placebo), the effectiveness of acupuncture has been proven. Therefore, we chose loratadine as the control group intervention in this trial so as to compare the differences between them, whether warm needling has an equal effect or is better than loratadine. Since western medicine is clearly being provided to the control group, with warm needling to the treatment group, it is difficult for us to achieve blinding of the participants and operators. Therefore, we chose blinding of the outcome assessors and the statistical analysts. To reduce the potential bias, strict random allocation concealment will be applied to minimize the differences between the two groups.

In winter, the cold climate, especially in the morning, is the commonest trigger of AR [32]. In TCM, AR is differentiated as a deficiency of *Wei Qi*, caused by external Wind and Cold; due to this pathogenesis of AR, warm needling at *Dazhui* (GV14) was included in the treatment. Moxibustion plays an important role in the prevention and treatment of disease [33], and previous research has reported that moxibustion has a positive effect on RA [34]. Professor Tian Conghuo, the distinguished veteran doctor of TCM, has established the therapeutic method as strengthening *yang* and expelling Cold for AR. In the treatment, warm needling was applied at *Dazhui* (GV14), combining with acupuncture at local acupoints. *Dazhui* (GV14) is the confluent acupoint of the three *yang* meridians of hand and foot and the Governor Vessel. Hence, this point is also named as “*yang* within *yang*.” Moxibustion at *Dazhui* (GV14) acts by invigorating *yang qi* of all the *yang* meridians, promoting the circulation in the Governor Vessel, expelling Cold and consolidating the defensive *qi*. Therefore, this acupoint is especially suitable for the treatment of allergic rhinitis, asthma, common cold, and the other disorders caused by *yang* deficiency [35].

In the preliminary experiment, we conducted a pilot RCT with a small sample size. The purpose of this pilot study was to test the feasibility of the trial. Participants were divided into three groups and accepted warm needling on ginger, acupuncture, and loratadine separately, 20 cases in each groups. The results showed that warm needling achieved a more persistent effect than acupuncture alone and loratadine alone [27]. As a result, this multicenter RCT was designed to prove the effect of warm needling on AR. Considering the complication of manipulation of warm needling on ginger, we replaced ginger by increasing the numbers of moxa sticks. The TNSS at 6-month follow-up was taken as the primary outcome in this trial.

A previous systematic review of 13 studies showed that acupuncture was effective at reducing PAR symptoms and improving patients’ quality of life [36]. However, the duration of observation in the included studies ranged from 4 weeks to 3 months, and the time of follow-up was short, resulting in lack of adequate evaluation of the long-term efficacy of acupuncture and moxibustion. Therefore, the time of follow-up in our study will be 6 months to ensure persistent observation of the efficacy maintenance.

We expect that this study results will provide evidence to determine the effects of warm needling therapy compared to loratadine. Our final goal of this study is to evaluate differences in the short-term and long-term effects between the two therapeutic methods, especially the long-term effect of warm needling therapy.

## Trial status

We are beginning to recruit participants for this trial. The trial is designed to be completed by 31 December 2017.

## Abbreviations

CACMS, China Academy of Chinese Medical Sciences; PAR, persistent allergic rhinitis; RCT, randomized controlled trial; RQLQ, Rhinoconjunctivitis Quality of Life Questionnaire; TCM, Traditional Chinese Medicine; TNSS, Total Nasal Symptom Score; TNNSS, Total Non-nasal Symptom Score

## Additional file

Additional file 1: SPIRIT 2013 checklist: recommended items to address in a clinical trial protocol and related documents. (DOC 80 kb)

## References

[1] Bousquet J, Van CP, Khaltaev N (2001). Allergic rhinitis and its impact on asthma. J Allergy Clin Immunol.

[2] Bousquet J, Khaltaev N, Cruz AA, Denburg J, Fokkens WJ, Togias A (2008). Allergic Rhinitis and its Impact on Asthma (ARIA) 2008 update. Allergy.

[3] Bousquet PJ, Leynaert B, Neukirch F, Sunyer J, Janson CM, Anto J (2008). Geographical distribution of atopic rhinitis in the European Community Respiratory Health Survey. Allergy.

[4] Han DM, Zhang L, Huang D, Wu YF, Dong Z, Xu G (2007). Self-reported prevalence of allergic rhinitis in eleven cities in China. Chin J Otorhinolaryngol Head Neck Surg.

[5] Zheng M, Wang XD, Bo MY, Wang KJ, Zhao Y, He F (2015). Prevalence of allergic rhinitis among adults in urban and rural areas of China: a population-based cross-sectional survey. Allergy Asthma Immunol Res.

[6] Christian JA, Christina J, Matthew H, Helen S (2012). Validity of two common asthma-specific quality of life questionnaires: Juniper mini asthma quality of life questionnaire and Sydney asthma quality of life questionnaire. Health Qual Life Outcomes.

[7] Schramm B, Ehlken B, Smala A, Quednau K, Berger K, Nowak D (2003). Cost of illness of atopic asthma and seasonal allergic rhinitis in Germany: 1-yr retrospective study. Eur Respir J.

[8] Simoens S (2012). The cost-effectiveness of immunotherapy for respiratory allergy: a review. Allergy.

[9] Nathan RA (2009). Management of patients with allergic rhinitis and asthma: literature review. South Med J.

[10] Pampura AN, Papadopoulos NG, Spičák V, Kurzawa R (2011). Evidence for clinical safety, efficacy, and parent and physician perceptions of levocetirizine for the treatment of children with allergic disease. Int Arch Allergy Immunol.

[11] Durham SR, Walker SM, Varga EM, Jacobson MR, Brien F, Noble W (1999). Long-term clinical efficacy of grass-pollen immunotherapy. N Engl J Med.

[12] Winther L, Arnved J, Malling HJ, Nolte H, Mosbech H (2006). Side-effects of allergen-specific immunotherapy: a prospective multi-centre study. Clin Exp Allergy.

[13] Choi SM, Park JE, Li SS, Jung H, Zi M, Kim TH (2013). A multicenter, randomized, controlled trial testing the effects of acupuncture on allergic rhinitis. Allergy.

[14] Yang ZX, Zhao YX (2002). Study on strengthening mice immunity and antineoplastic function of moxibustion. Chin Arch Tradit Chin Med.

[15] Su L, Li L, Yang JS, Zhu B (2010). Modern research on the effect of moxibustion on body function. Chin J Inf Tradit Chin Med.

[16] Seidman MD, Gurgel RK, Lin SY, Schwartz SR, Baroody FM, Bonner JR (2015). Clinical practice guideline: allergic rhinitis. Otolaryngol Head Neck Surg.

[17] Clissold SP, Sorkin EM, Goa KL (1989). Loratadine: a preliminary review of its pharmacodynamic properties and therapeutic efficacy. Drugs.

[18] Golightly LK, Greos LS (2005). Second-generation antihistamines: actions and efficacy in the management of allergic disorders. Drugs.

[19] Sonnemann U, Möller M, Bilstein A (2014). Noninterventional open-label trial investigating the efficacy and safety of ectoine containing nasal spray in comparison with beclomethasone nasal spray in patients with allergic rhinitis. J Allergy (Cairo).

[20] North ML, Walker TJ, Steacy LM, Hobsbawn BG, Allan RJ, Hackman F (2014). Add-on histamine receptor-3 antagonist for allergic rhinitis: a double blind randomized crossover trial using the environmental exposure unit. Allergy Asthma Clin Immunol.

[21] Juniper EF, Rohrbaugh T, Meltzer EO (2003). A questionnaire to measure quality of life in adults with nocturnal allergic rhinoconjunctivitis. J Allergy Clin Immunol.

[22] Juniper EF, Guyatt GH, Griffith LE, Ferrie PJ (1996). Interpretation of rhinoconjunctivitis quality of life questionnaire data. J Allergy Clin Immunol.

[23] Saverno KR, Seal B, Goodman MJ, Meyer K (2009). Economic evaluation of quality-of-Life improvement with second-generation antihistamines and montelukast in patients with allergic rhinitis. Am Health Drug Benefits.

[24] Perrin Y, Nutten S, Audran R, Berger B, Bibiloni R, Wassenberg J, et al. Comparison of two oral probiotic preparations in a randomized crossover trial highlights a potentially beneficial effect of Lactobacillus in patients with allergic rhinitis. Clin Transl Allergy. 2014;41. Published online 2014 January 6. doi:10.1186/2045-7022-4-1. 10.1186/2045-7022-4-1 PMC3925289 24393277

[25] Zhang L, Li L, Shi DZ, Chen LQ, Zheng KM, Cheng K (2015). Sphenopalatine ganglion stimulation with one acupuncture needle for moderate-severe persistent allergic rhinitis: study protocol for a multicenter randomized controlled trial. Trials.

[26] Catana IV, Chirila M, Negoias S, Bologa R, Cosgarea M (2013). Effects of corticosteroids on hyposmia in persistent allergic rhinitis. Clujul Med.

[27] Lin C, Tian N, Ye YM (2013). Ginger moxibustion and warm needling method on persistent allergic rhinitis: a randomized controllled trial. Beijing J Tradit Chin Med.

[28] Ellis AK, Zhu Y, Steacy LM, Walker T, Day JH (2013). A four-way, double-blind, randomized, placebo controlled study to determine the efficacy and speed of azelastine nasal spray, versus loratadine, and cetirizine in adult subjects with allergen-induced seasonal allergic rhinitis. Allergy Asthma Clin Immunol.

[29] Martins J, Sousa LM, Oliveira AS (2009). Recomendações do enunciado CONSORT para o relato de estudos clínicos controlados e randomizados. Medicina.

[30] Pherson HM, Altman DG, Hammerschlag R, Li Y, Taixiang W, White A (2010). Revised Standards for Reporting Interventions in Clinical Trials of Acupuncture (STRICTA): extending the CONSORT Statement. PLoS Med.

[31] Piaggio G, Elbourne DR, Ahman DG (2006). Reporting of noninferiority and equivalence randomized trials: an extension of the CONSORT statement. JAMA.

[32] Li CW, de Chen H, Zhong JT, Lin ZB, Peng H, Lu HG (2014). Epidemiological characterization and risk factors of allergic rhinitis in the general population in Guangzhou City in China. PLoS One.

[33] Liu Q, Yang J, Zhao BX (2014). Overview of different moxibustion methods treating allergic rhinitis. World Chin Med.

[34] Chen S, Guo S, Wang J, Ha E, Marmori F, Wang Y (2015). Effectiveness of moxibustion for allergic rhinitis: protocol for a systematic review. BMJ Open.

[35] Li QY (2000). The experience of Professor Tian Conghuo applying moxibustion at *Dazhui*. Chin J Clin.

[36] Feng SY, Han MM, Fan YP, Yang GW, Liao ZP, Liao W (2015). Acupuncture for the treatment of allergic rhinitis: a systematic review and meta-analysis. Am J Rhinol Allergy.

